# Vascular retinal findings after COVID-19 vaccination in 11 cases: a coincidence or consequence?

**DOI:** 10.5935/0004-2749.20220071

**Published:** 2025-08-21

**Authors:** Letícia S. C. da Silva, Luciana P. S. Finamor, Gabriel C. Andrade, Luiz H. Lima, Claudio Zett, Cristina Muccioli, Eduardo P. Sarraff, Paula M. Marinho, Julia Peruchi, Raiza D. de L. Oliveira, Lena Giralt, Ivonne Charcan, Alex Fonollosa, Jose D. Diaz, Janet L. Davis, Heloisa Nascimento, Rubens Belfort Jr

**Affiliations:** 1 Department of Ophthalmology, Universidade Federal de São Paulo, São Paulo, SP, Brazil; 2 Pontificia Universidad Católica de Valparaíso, Valparaíso, Chile; 3 Instituto Paulista de Estudos e Pesquisa em Oftalmologia, Instituto da Visão, São Paulo, SP, Brazil; 4 Visão Center, São Mateus, ES, Brazil; 5 Department of Ophthalmology, Universidade de São Paulo, São Paulo, SP, Brazil; 6 Department of Ophthalmology, BioCruces Bizkaia Health Research Institute, Cruces University Hospital, University of the Basque Country, Barakaldo, Spain; 7 Bascom Palmer Eye Institute, University of Miami Miller School of Medicine, Miami, FL, USA; 8 Hospital Municipal de Barueri, Barueri, SP, Brazil

**Keywords:** COVID-19, Coronavirus infection, Vaccine, Arterial occlusion, Venous occlusion, Susac syndrome, COVID-19, Infecções por coronavírus, Vacina, Oclusão arterial, Oclusão venosa, Síndrome de Susac

## Abstract

**Purpose:**

The primary purpose of this study was to assess vascular retinal findings temporally related to COVID-19 vaccination. With greater information regarding all possible future adverse events, we hope to understand the real dimension and relevance of what was presented.

**Methods:**

Eleven patients with visual complaints after COVID-19 vaccination were enrolled. Data on the following were included: age, sex, vaccine, time of symptom onset, systemic findings, medical history, best-corrected visual acuity, and ocular findings by slit-lamp biomicroscopy as well as multimodal retinal imaging (color fundus, red-free photography, spectral-domain optical coherence tomography, optical coherence tomography angiography, and fluorescein-angiography). Inclusion criteria were the presence of ophthalmologic signs within 30 days after the first or second dose of any COVID-19 vaccine.

**Results:**

Of 11 patients, five had arterial occlusion (45.4%), four had venous occlusion (36.4%), and two (18.2%) had nonspecific vascular alterations suggestive of retinal ischemia such as cotton-wool spots. The mean age was 57 (SD = 16; range: 27-84) years. The mean time of symptoms onset was 10 (SD = 5.4; range: 3-16) days. Nine patients were female (81.8%). Systemic risk factors were observed in 36.4% of patients. Two patients had both neurological and visual symptoms, with arterial occlusion. Overall, 36.4% patients had COVID-19 in the previous year. Seven patients (63.6%) received ChAdOx1 nCoV-19 (AZD1222) vaccine.

**Conclusions:**

Our data suggest that retinal events temporally related to COVID-19 vaccination are possible but are very rare. The relationship of these events with post-COVID-19 vaccination warrants further attention to derive a meaningful conclusion.

## Abstract (pt)

**Objetivos:**

o principal objetivo deste estudo foi descrever pacientes com achados vasculares retinianos temporalmente relacionados à vacinação contra COVID-19. Com maior notificação de possíveis eventos adversos similares, esperamos compreender a real dimensão e relevância do que foi apresentado. **M**é**todos:** Onze pacientes com queixas visuais após vacinação contra COVID-19 foram estudados. Os dados analisados foram: idade, gênero, tipo de vacinação, tempo de aparecimento de sintomas, achados sistêmicos, antecedentes pessoais, acuidade visual com melhor correção, biomicroscopia e imagem retiniana multimodal (retinografia colorida, red-free, SD-OCT, OCTA e angiofluoresceinografia). Os critérios de inclusão foram a presença de alterações oftalmológicas ocorridas dentro de 30 dias após a primeira ou segunda dose de qualquer vacina contra COVID-19.

**Resultados:**

Onze pacientes foram incluídos: 5 com oclusão arterial (45,4%), 4 com oclusão venosa (36,4%) e 2 (18,2%) com alterações não específicas vasculares sugestivas de isquemia retiniana como exsudatos algodonosos. A idade média dos pacientes foi de 57 anos (DP=16; com intervalo de 27 a 84 anos). A média de tempo de aparecimento de sintomas após a vacinação foi de 10 dias (DP=5,4; com intervalo de 3 a 16 dias). Nove dos onze pacientes eram do sexo feminino (81,8%). Fatores de risco sistêmicos foram observados em 36,4% dos pacientes. Dois pacientes tiveram sintomas neurológicos e visuais, com oclusão arterial. 36,4% dos pacientes tiveram infecção prévia por COVID-19 no último ano. Sete pacientes (63,6%) receberam a vacina ChAdOx1 nCoV-19 (AZD1222).

**Conclusões:**

nossos dados sugerem que eventos retinianos temporalmente relacionados à vacinação contra COVID-19 são possíveis, porém raros. A relação entre estes eventos pós-vacinais exigem futura atenção antes de maiores conclusões.

## INTRODUCTION

Severe acute respiratory syndrome coronavirus-2 (SARS-CoV-2) has infected millions of people globally, causing the coronavirus disease 2019 (COVID-19) pandemic. This has resulted in an unprecedented effort to develop vaccines against this virus. As vaccines are now being introduced globally, we face the prospect of millions of people being vaccinated with multiple vaccine types, many of which use new platforms.

Although highly effective and well-tolerated in most patients, immunization is not without side effects. The rates of mild acute adverse events reported in vaccine registration trials typically range from 10% to 30%^(1)^.

Few studies have reported vein and artery retinal occlusion, uveitis, acute idiopathic maculopathy, acute macular neuroretinopathy, Vogt-Koyanagi-Harada disease, and multiple evanescent white dot syndrome after administration of different vaccines, such as those for B hepatitis, yellow fever, smallpox, Influenza, Neisseria meningitidis, and Herpes Zoster^(2-13)^.

Recently, Fowler et. al. reported a case of a 33-year-old male who presented with unilateral central serous retinopathy 3 days after mRNA BNT162b2 vaccine administration (Pfizer/BioNTech)^(14)^. Mudie et al. reported a case of a 43-year-old female with asymptomatic COVID-19 infection who developed panuveitis 3 days after her second dose of mRNA BNT162b2 vaccine (Pfizer/BioNTech)^(15)^. In addition, Book at al. reported bilateral paracentral scotomas in a 21-year-old woman 3 days after receiving her first ChAdOx1 nCoV-19 (AZD1222) vaccine (Oxford/AstraZeneca)^(16)^.

This study describes a series of patients with vascular retinal findings, temporally associated with COVID-19 vaccines, including inactivated SARS-CoV-2 vaccine (CoronaVac), mRNA-1273 vaccine (Moderna), mRNA BNT162b2 (Pfizer/BioNTech), and ChAdOx1 nCoV-19 (AZD1222) vaccine (Oxford/AstraZeneca).

## METHODS

In March 2021, a study group of ocular adverse events after SARS-CoV-2 vaccination was created in São Paulo, Brazil, following surveillance and exchange of information. Eleven patients with visual complaints after COVID-19 vaccine were studied. Inclusion criteria were as follows: presence of ophthalmologic signs within 30 days after the first or second dose of any COVID-19 vaccine.

Data on the following were collected: age, sex, type of vaccine received, time of symptom onset after vaccination, presence or absence of systemic findings, medical history (including previous COVID-19 infection), best-corrected visual acuity, and ocular findings by slit-lamp biomicroscopy as well as multimodal retinal imaging (color fundus, red-free photography, SD-OCT, OCTA, and fluorescein-angiography). We analyzed cases from March to August 2021 that were reported by specialists from Brazil, USA, and Spain.

The study was approved by the Health Ethics Committee, and patients agreed to participate.

## RESULTS

The mean age of patients was 57 (SD = 16; range: 27-84) years. The mean time of symptom onset after vaccination was 10 (SD = 5.4, range: 3-16) days. Nine of eleven patients were female (81.8%).

**Figure 1:**
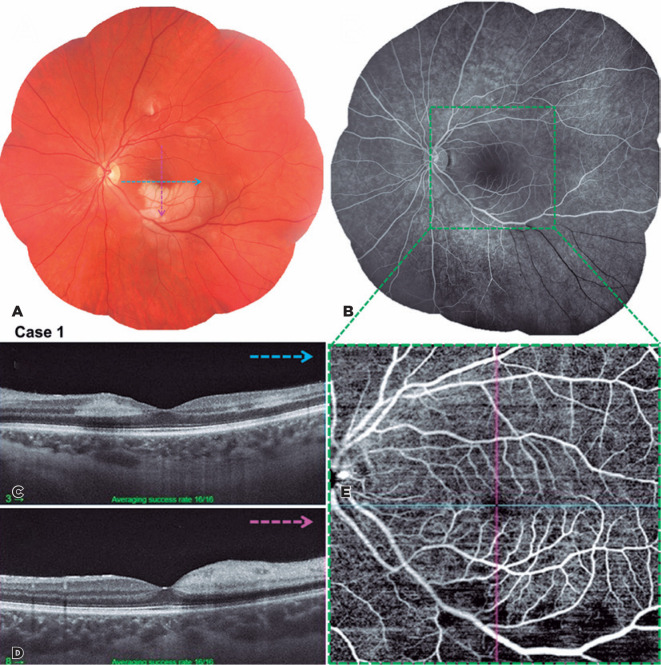
(A-E) Case 1. Multimodal imaging of study patient’s left eye at presentation. A. Color photograph of the left eye demonstrates inferior perifoveal pallor of the macular region, corresponding to focal retinal artery occlusion. (B) Angiography of the left eye demonstrates widening of the foveal avascular zone. (C and D) Swept-Source optical coherence tomography of the left eye shows a hyperreflective band at the level of the inner nuclear layer and outer plexiform layer with attenuation of the underlying inner segment/ outer segment (IS/OS), and OS/ retinal pigment epithelium layers (C) and thickening of the inner retinal layers with shadow effect on the outermost retinal layer (D), corresponding with pericentral acute middle maculopathy associated with central retinal artery occlusion. (E) OCT angiography reveals decreased capillary density in the macular region, corresponding to previously described findings.

Systemic risk factors were observed in 36.4% of patients. In addition, 36.4% patients had COVID-19 infection in the previous year. Two patients had both neurological and visual symptoms with arterial occlusion, and details on one of them are presented in figure 1.

**Figure 2:**
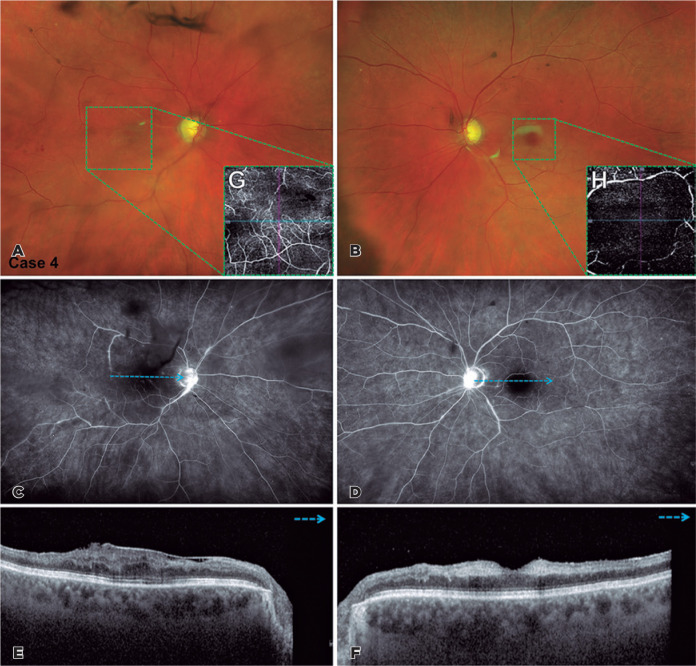
**(A-H)** Case 4. Multimodal imaging of patient’s both eyes at presentation. (A and B) Color photograph of both eyes demonstrates diffuse pallor of the macular region, corresponding to central retinal artery occlusion. (C and D) Angiography of both eyes demonstrates hyperfluorescence due to papillary leakage associated with the areas of vasculitis and widening of the foveal avascular zone. (E and F) Spectral-domain optical coherence tomography of the right eye (E) shows the epiretinal membrane associated with the disorganization of intraretinal structures. Left eye (F) revealed thickening of the inner retinal layers with shadow effect on the outermost retinal layers. (G and H) OCT angiography reveals decreased capillary density in the macular region, corresponding to previously described findings.

**Figure 3:**
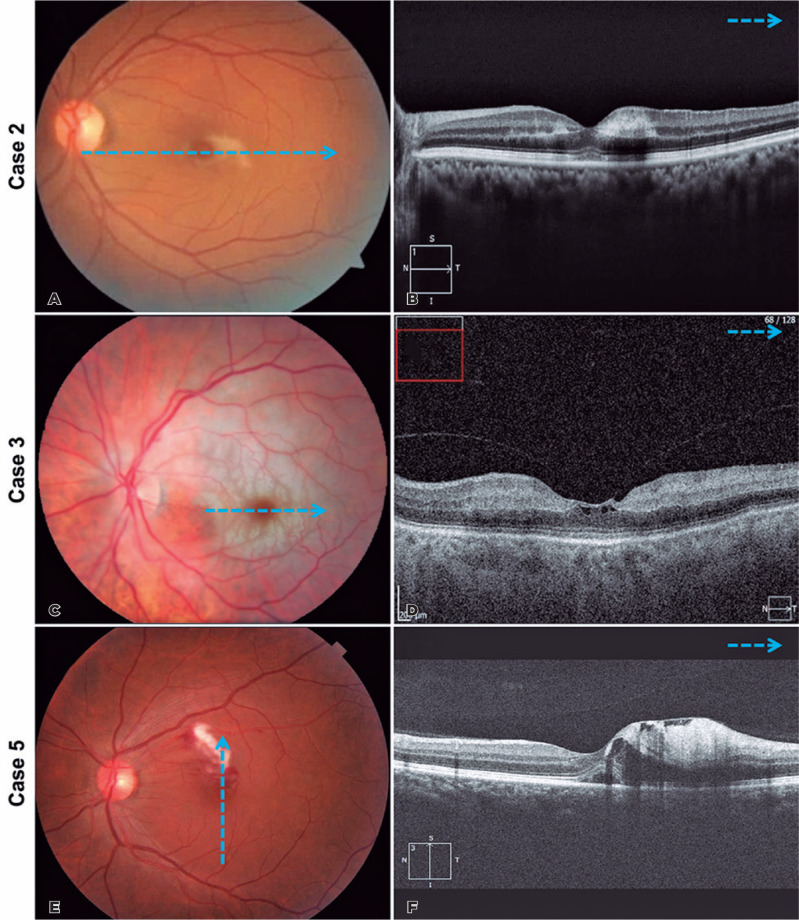
(A and B) Case 2. Multimodal imaging of patient’s left eye at presentation. (A). Color photograph of the left eye demonstrates perifoveal pallor of the macular region, corresponding to the focal retinal artery occlusion. (B) Spectral-domain optical coherence tomography of the left eye revealed a hyperreflective band at the level of the inner nuclear layer and outer plexiform layer with attenuation of the underlying inner segment/outer segment (IS/OS), and OS/retinal pigment epithelium layers. (C and D) Case 3. Multimodal imaging of the patient’s left eye at presentation. (C) Color photograph of the left eye demonstrates diffuse pallor of the macular region with a “cherry macula” appearance, corresponding to central retinal artery occlusion. (D) Spectral-domain optical coherence tomography of the left eye shows thickening of the inner retinal layers with shadow effect on the outermost retinal layers. (E and F) Case 5. Multimodal imaging of the patient’s left eye at presentation. (E) Color photograph of the left eye demonstrates both venous and arterial macular branch occlusions with macular edema. (F) Spectral-domain optical coherence tomography of the left eye shows hyperreflectivity in retinal nerve fiber layer, corresponding to intraretinal hemorrhages and cysts in the inner nuclear layer as well as smaller perifoveal cysts associated with sub-retinal fluid.

**Figure 4:**
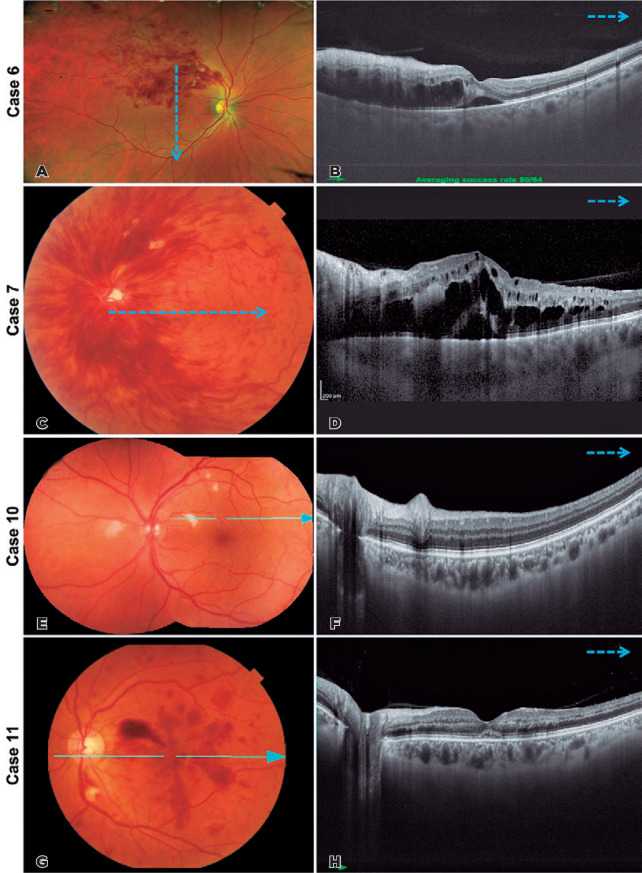
(A and B) Case 6. Multimodal imaging of the patient’s right eye at presentation. (A) Color photograph of the right eye demonstrates superotemporal intraretinal hemorrhages with macular edema, corresponding to branch retinal vein occlusion. (B) Swept-source optical coherence tomography of the right eye shows large central cysts in the inner nuclear layer and smaller perifoveal cysts associated with sub-retinal fluid. (C and D) Case 7. Multimodal imaging of the patient’s left eye at presentation. (C) Color photograph of the left eye demonstrates diffuse intraretinal hemorrhages emerging from the optic disc with macular edema corresponding to central retinal vein occlusion. (D) Swept-source optical coherence tomography of the left eye shows large central cysts in the inner nuclear layer and smaller perifoveal cysts associated with sub-retinal fluid. (E and F) Case 10. Multimodal imaging of the patient’s left eye at presentation. (E) Color photograph of the left eye demonstrates intraretinal hemorrhages. (G and H) Case 11. Multimodal imaging of the patient’s left eye at presentation. (G) Color photograph of the left eye demonstrates intraretinal hemorrhages within the posterior pole and a cotton-wool spot inferior to the optic disc. (H) Spectral-domain optical coherence tomography of the left eye shows hyperreflectivity in the retinal nerve fiber layer, corresponding to intraretinal hemorrhages and sub-retinal fluid.

Among the 11 described cases, five had arterial occlusion (45.4%) (Figures 1, 2, and 3), four had venous occlusion (36.4%), and two (18.2%) had nonspecific vascular alterations, suggestive of retinal ischemia such as cotton-wool spots (Figure 4). The most frequently administered vaccine was ChAdOx1 nCoV-19 (AZD1222), with 7 of 11 or 63.6% of patients receiving it. The collected data are summarized in table 1.

**Table 1:** Summary of demographic, epidemiological, and clinical characteristics of all patients

Case	Sex	Age	City/country	Vaccine administered	Symptoms onset after vaccination (days)	Systemic findings	Type of retinal manifestation	Systemic risk factors	Previous COVID-19
**1**	Female	27	São Paulo,	Coronavac,	14	Mental confusion,	Arterial	None	Yes (COVID-19
			Brazil	Sinovac		amnesia, paresthesia,			1 year ago)
						tinnitus, cranial RMI			
						with hypersignal in			
						white matter			
**2**	Female	57	São Paulo, Brazil	Coronavac, Sinovac	15	Hear loss, headache, leptomeningitis, bilateral cochlear hearing loss	Arterial	None	Yes (COVID-193 months ago)
**3**	Female	84	Espírito Santo,	Oxford/	16	None	Arterial	Systemic	No
			Brazil	AstraZeneca				arterial	
								hypertension	
								and bilateral	
								carotid	
								atherosclerosis	
**4**	Female	74	Miami, Florida,	Moderna	3	None	Arterial	None	No
			USA						
**5**	Female	39	São Paulo,	Oxford/	3	None	Arterial	Psoriasis	Yes (COVID-19
			Brazil	AstraZeneca				with previous	1 year before)
								use of	
								methotrexate	
**6**	Female	66	São Paulo,	Oxford/	16	Headache	Venous	Hysterectomy	No
			Brazil	AstraZeneca				4 months	
								ago due to	
								endometrial	
								hypertrophy,	
								increased	
								BMI, and	
								increased	
								apoliprotein a	
**7**	Male	51	Bilbao, Spain	Pfizer	6	None	Venous	None	Yes
									(COVID-19 10
									months ago)
**8**	Male	66	São Paulo,	Oxford/	4	None	Venous	Systemic	No
			Brazil	AstraZeneca				arterial	
								hypertension	
**9**	Female	54	São Paulo,	Oxford/	10	None	Venous	None	No
			Brazil	AstraZeneca					
**10**	Female	56	São Paulo,	Oxford/	10	None	Non-perfusion	None	No
			Brazil	AstraZeneca					
**11**	Female	50	São Paulo,	Oxford/	15	None	Non-perfusion	None	No
			Brazil	AstraZeneca					

## DISCUSSION

Although it may seem intuitive that agents intended to activate the immune system may lead to the development of unintended immune activation and inflammatory adverse events in some patients, the precise mechanisms underlying such events is still unclear^(17)^.

Acute retinal vascular occlusions are common causes of visual impairment. Retinal artery and vein occlusions are associated with increased age and cardiovascular risk factors^(18)^. The presence of cotton-wool spots represents nerve fiber layer infarctions that result from inner retinal ischemia, secondary to the occlusion of precapillary arterioles, and can occur in several systemic diseases^(19)^.

In this study, we found retinal vascular findings in 11 patients after COVID-19 vaccination, of which 7 (63.6%) had no previous vascular risk factors. The prevalence of retinal vein occlusions in predominantly white populations is 0.6%-1.2% (BRVO) and 0.1%-0.4% (CRVO), with an incidence of 0.12% (BRVO) and 0.03% (CRVO) per year. The estimated incidence of acute CRAO is 1-2 per 100,000 people per year or 0.001%^(18)^.

Although rare, retinal vasculitis has also been reported as an adverse event following Influenza vaccination, mainly in elderly and female patients. This adverse event is probably associated with an increase in post-vaccine proinflammatory cytokines^(7)^.

In the present study, two women with arterial occlusion had neurological symptoms, hearing disorders, and magnetic resonance imaging findings suggestive of Susac syndrome; one had bilateral retinal occlusion. Both had received the second dose of inactivated SARS-CoV-2 vaccine (CoronaVac) just 7 and 15 days prior to visual symptoms, and both had a history of COVID-19 infection. It is unknown if COVID-19 history increases the risk of side effects from the vaccine.

Vaccination as a trigger for immunologically related vascular inflammation has been previously described. Landa et al. described a case of an adult male who developed multiple branch retinal arteriolar occlusions and encephalopathy that occurred 10 days after smallpox vaccination. This was hypothesized to be Susac syndrome induced by vaccination^(2)^.

This case series has some limitations. We described patients from different countries (Brazil, USA, and Spain) who received different vaccines. The mRNA-1273 (Moderna) and mRNA BNT162b2 (Pfizer/BioNTech) vaccines are based on mRNA that encodes a SARS-CoV-2 spike protein, whereas the CoronaVac (Sinovac Life Sciences, Beijing, China) comprises inactivated SARS-CoV-2 virus. In contrast, the ChAdOx1 nCoV-19 (AZD1222) vaccine (Oxford/AstraZeneca) uses a chimpanzee adenovirus-based vector. The most common vaccine among the patients was ChAdOx1 nCoV-19 (AZD1222) vaccine (Oxford/AstraZeneca), but the small sample size and availability of different vaccines among centers limited our ability to link the retinal events to a specific vaccine.

Antigenic cross-reactions, immediate hypersensitivity, and deposition of immune complexes directly related to the vaccine are hypothesized as explanations for the pathophysiology of retinal vascular occlusions after COVID-19 vaccination; however, there is no confirmed mechanism^(3)^. Another theory implicates adjuvants that enhance the immunogenicity of vaccine antigens and may increase the risk of autoimmune side effects^(20)^. The mRNA-1273 (Moderna) and mRNA BNT162b2 (Pfizer/ BioNTech) vaccines use lipid nanoparticles, whereas the inactivated SARS-CoV-2 vaccine (CoronaVac) uses aluminum as adjuvants. The ChAdOx1 nCoV-19 (AZD1222) vaccine (Oxford/AstraZeneca), which was more common among our patients, does not comprise adjuvants.

There were two cases of retinal hemorrhages and cotton-wool spots, occurring 10 and 15 days, respectively, after the first dose of Oxford-AstraZeneca vaccine, both in women. Reportedly, retinal hemorrhages occur in children after routine vaccinations^(21)^, and cotton-wool spots occur after smallpox vaccination^(2)^. Natural COVID-19 infection has also been reported to be associated with retinal and vitreous abnormalities, such as cotton-wool spots, outer retina changes, and vitritis^(22,23)^.

Although we cannot exclude the fact that the retinal events were coincidental and related to systemic risk factors in 4 of 11 (36.4%) patients, there was a very strong relationship between vaccination and retinal vascular events at a mean of 10 days after vaccination. The COVID-19 pandemic is propelling the global vaccine number to an unprecedented level. It is possible that we will have an increasing number of ocular events, which may or may not be caused or not by vaccination, and it is of great importance to describe new findings. To the best of our knowledge, no other study has described cases of such retinal manifestations following COVID-19 vaccination in the literature. Only future observation of an increase in the incidence of these events, through robust epidemiological surveys, will help better elucidate the causality of these findings with COVID-19 vaccination.

The speed of global vaccination against COVID-19 is unprecedented. Our data suggest that retinal events with a temporal association after COVID-19 vaccination are possible but very rare. We alert ophthalmologists and clinicians about the need to investigate suspected cases and carefully search for preexisting conditions that might explain the retinal vascular events, so that treatment can be arranged if needed. In addition, reporting rare events to central agencies such as the Vaccine Adverse Event Reporting System in the United States and Brazilian Health Regulatory Agency can help supply required epidemiologic evidence regarding whether these vascular events are more common after COVID-19. The immunologic basis of vaccine-related retinal vascular events deserves further exploration.
